# Shortness of Breath: An Unusual Presentation of Bladder Injury. A Case Report and Literature Review of Urinothorax

**DOI:** 10.7759/cureus.4559

**Published:** 2019-04-28

**Authors:** Ahmad Ramahi, Kanana Mohammad Aburayyan, Ali Alqahtani, Tamer S Said Ahmed, Mohammad Taleb

**Affiliations:** 1 Internal Medicine, University of Toledo Medical Center, Toledo, USA; 2 Pulmonary / Critical Care Medicine, University of Toledo Medical Center, Toledo, USA

**Keywords:** urinothorax, transudative pleural effusion, bladder injury

## Abstract

Urinothorax is the presence of urine in the pleural space. It can occur at any age and is more common in males. It typically results from obstructive uropathy but can also be caused by malignancy or trauma. Urinothorax is a rare cause of transudative pleural effusion and the only cause of low pH (pH <7.4) transudative effusion. We present the case of a 51-year-old female patient who had recently undergone a urological procedure and came to the emergency department reporting shortness of breath. A chest X-ray revealed a newly developed, large, right-sided pleural effusion. Thoracentesis yielded a transudative yellow fluid of normal pH with a creatinine-to-serum creatinine ratio of 1.7. A computed tomography (CT) cystogram showed extravasated contrast material within the pelvis, from which a diagnosis of urinothorax was confirmed and treated. Urinothorax is a rare diagnosis that requires a multidisciplinary treatment approach, usually including a pulmonologist and a urologist. After the genitourinary disease is treated, the urinothorax usually resolves.

## Introduction

In simple terms, urinothorax is the presence of urine in the pleural space. First described by Corriere et al. in 1968 [1], urinothorax can occur at any age and is more common in males [2]. It may result from obstructive uropathy and typically occurs as an ipsilateral pleural effusion with an obstructed kidney. However, contralateral or bilateral urinothoraces have also been reported in the literature. Urinothorax may also result from genitourinary (GU) malignancy or trauma, including iatrogenic from urologic procedures [3]. It is a rare cause of transudative pleural effusion [4] and one of two causes of low pH (pH <7.4) transudative pleural effusion [4]. We report a 51-year-old female patient who presented to the emergency department with a chief concern of shortness of breath and a newly developed, large, right-sided pleural effusion shortly after undergoing a urological procedure.

## Case presentation

A 51-year-old female patient presented to the hospital, reporting shortness of breath of two days’ duration that was associated with a dry cough but not with fever or chills, abdominal pain, nausea or vomiting, or a change in bowel or urine habits. She had a medical history of hypertension, hypothyroidism, diabetes mellitus type 2, and breast cancer for which she had undergone a lumpectomy. Of note, the patient had taken tamoxifen for five years following her lumpectomy, after which she developed postmenopausal bleeding. As tamoxifen therapy increases the risk for endometrial cancer, an endometrial biopsy was done that showed atypical hyperplasia with atypia for which the patient underwent a total abdominal hysterectomy and bilateral salpingo-oophorectomy. The surgery was complicated by an accidental bladder injury that was repaired at the time of surgery, 15 days prior to her current admission.

On physical examination, the patient’s blood pressure was 114/76 mmHg, heart rate 93 beats per minute, and respiratory rate 20 breaths per minute. Her temperature was 36.1°C and oxygen saturation 94% on room air. She was alert and oriented to person, place, and time. Heart examination showed a regular rate and rhythm with normal S1 and S2 sounds. Lung examination showed decreased breath sounds with dullness on percussion of the right side of the chest. Her abdomen was soft and lax, with no tenderness or organomegaly. There were no other significant physical findings.

**Figure 1:**
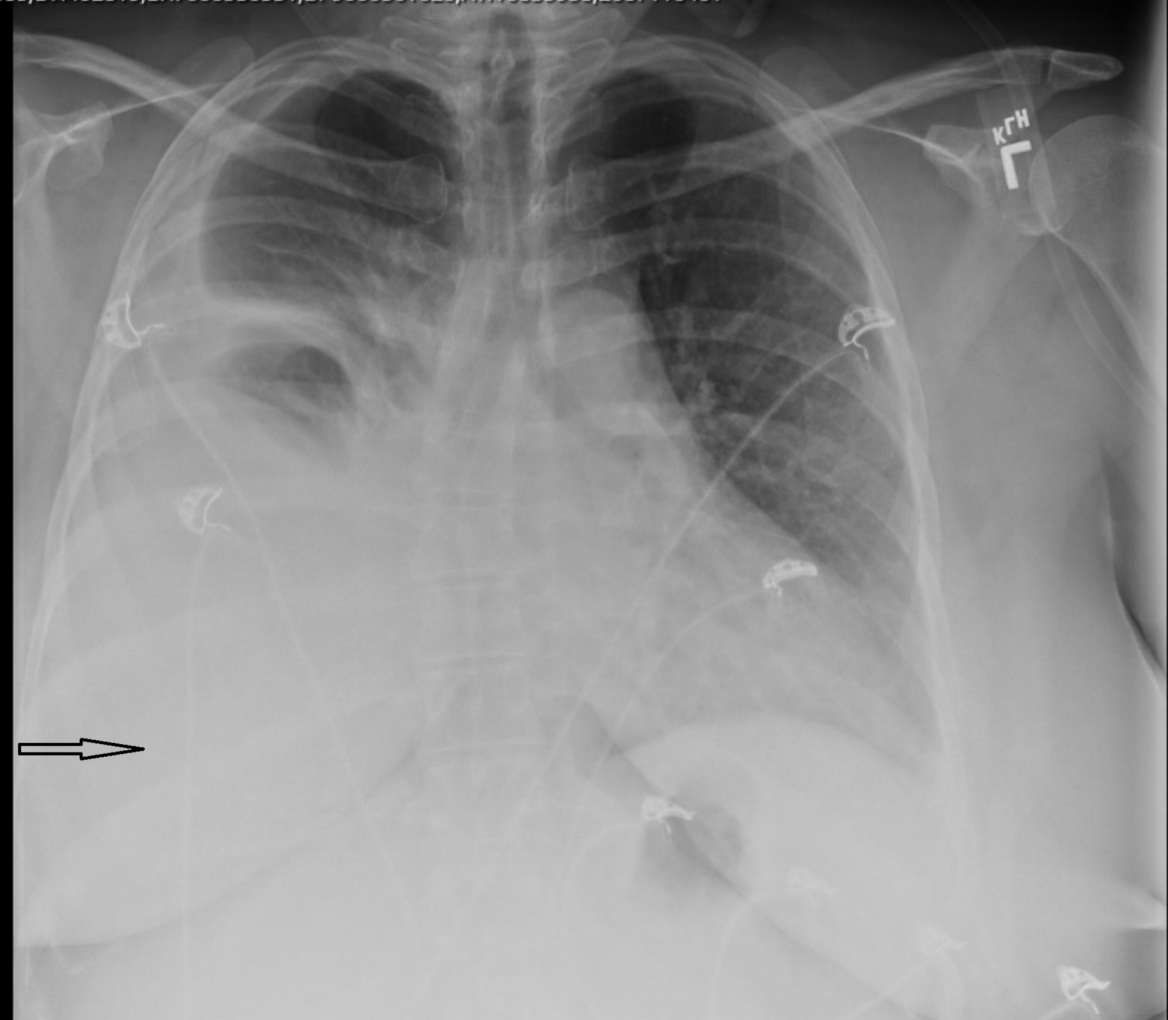
Chest X-ray posterior–anterior (PA) showing a right-sided pleural effusion (arrow)

Laboratory findings were as follows: leukocytosis with a white blood cell (WBC) count of 14000 /μL; hemoglobin level of 10 g/dL; serum creatinine level of 3.9 mg/dL (baseline 0.6 mg/dl); blood urea nitrogen level of 35 mg/dL; negative test results for antinuclear antibody, rheumatoid factor, and glomerular basement membrane antibody IgG; normal results for complement C3 and C4 levels; erythrocyte sedimentation rate 32 mm/h. The chest X-ray showed a large, right-sided pleural effusion (Figure 1). A transthoracic echocardiogram showed a preserved left ventricular ejection fraction with no valvular abnormalities.

The pulmonary team was consulted and the patient underwent thoracentesis that yielded 3 L of yellow fluid. She reported immediate improvement in shortness of breath after the procedure. Pleural fluid analysis (PFA) showed the fluid to be transudative in accordance with Light’s criteria, with the following values: lactate dehydrogenase (LDH) 143 U/L, albumin 1.6 g/dL, total protein 2.8 g/dL, glucose 115 mg/dL, pH 7.5, and triglycerides 45 mg/dL. Cytological examination of the aspirated fluid showed no malignant cells, and the results of fluid culture were negative. The serum LDH level was 290 U/L, total protein 7 g/dL, and albumin 3.9 g/dL. Because of the pleural fluid color and the patient’s recent procedure, pleural creatinine was measured and found to be 6.72 mg/dL, with a pleural fluid creatinine-to-serum creatinine ratio of 1.7, suggesting a diagnosis of urinothorax.

**Figure 2:**
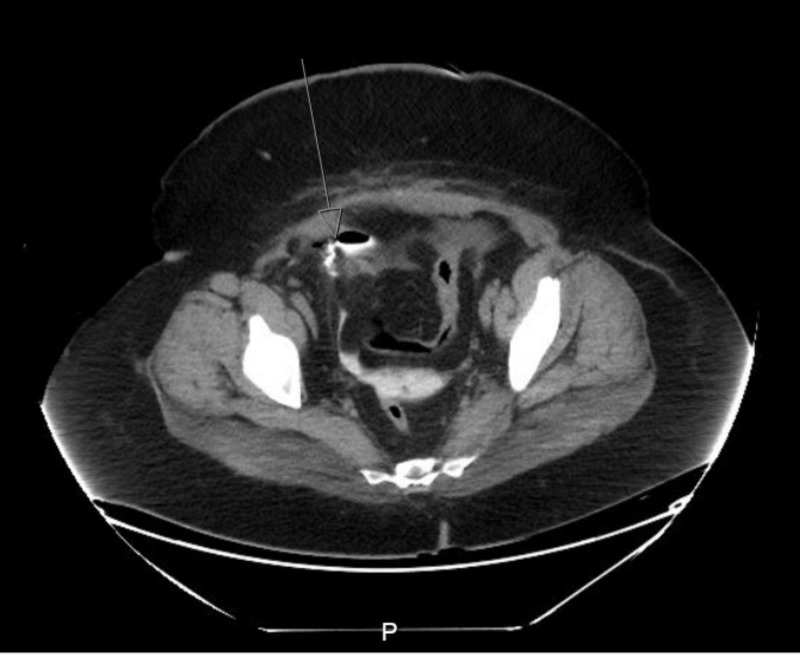
CT cystogram showing extravasated contrast within the pelvis (arrow)

A computed tomography (CT) scan of the abdomen and pelvis with contrast was performed, which revealed a small amount of subdiaphragmatic and perihepatic fluid, as well as fluid in the cul-de-sac. The urology team was consulted and a CT cystogram was performed, showing extravasated contrast material within the pelvis, with a small pocket of contrast noted along the right anterior aspect of the urinary bladder (Figure 2), likely at the site of the bladder leak. A renal ultrasound (US) showed no hydronephrosis, mild bilateral renal cortical thinning, and a small cyst in the superior left kidney.

**Figure 3:**
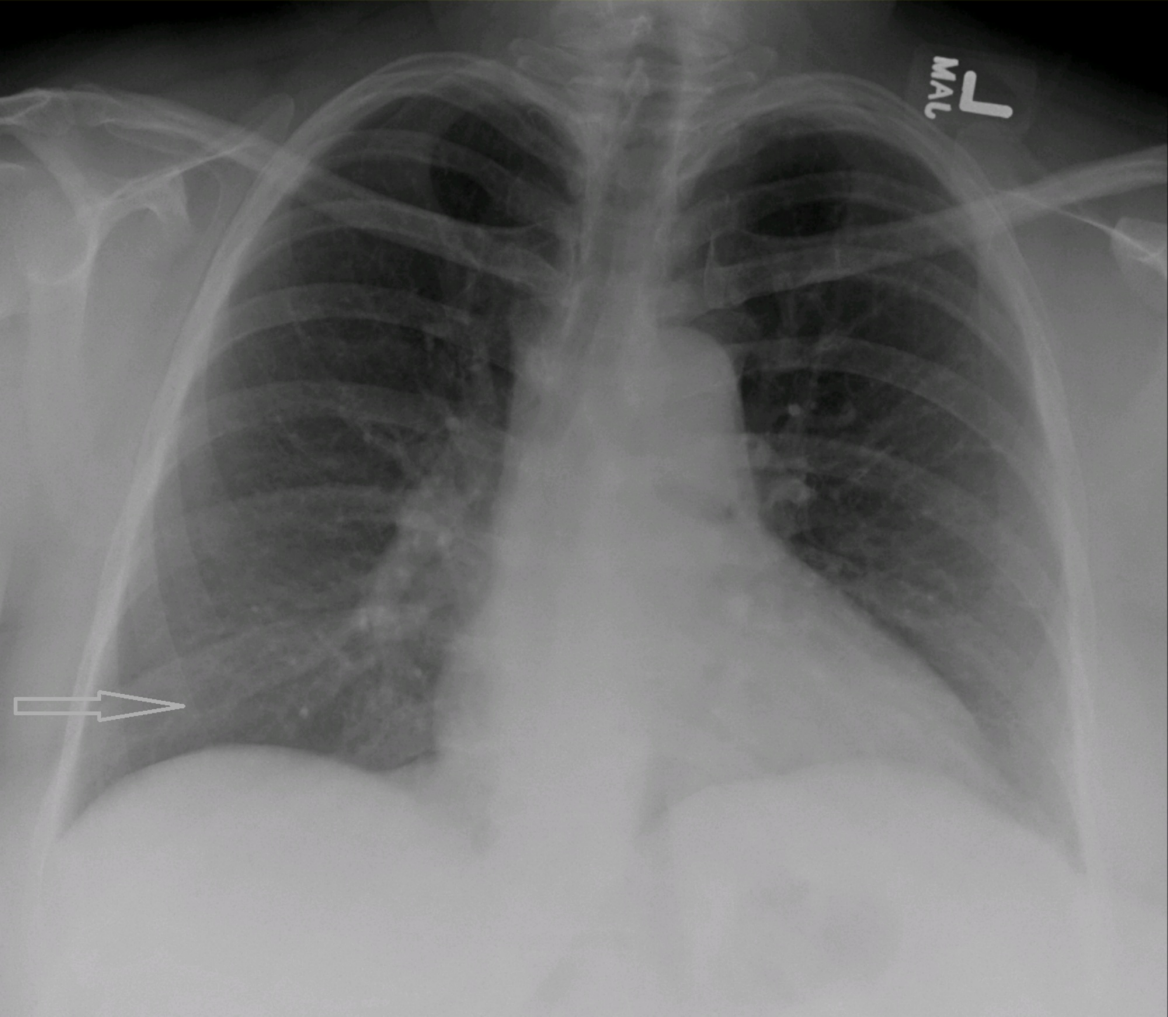
Chest X-ray showing the resolution of the right-sided pleural effusion (arrow)

With the source of the urine leak from the genitourinary (GU) tract established, together with a pleural effusion creatinine level of 6.72 mg/dL and a pleural fluid/serum creatinine (PF/S Cr) ratio of >1, we confirmed the diagnosis of urinothorax. A Foley’s catheter was used to seal the bladder leak, and a repeated chest X-ray one month later showed resolution of the right-sided pleural effusion (Figure 3), after which the patient reported no further shortness of breath. She was discharged with the Foley’s catheter in place and instructions to follow up with urology in two weeks. Repeated fluoroscopy cystography on follow-up showed no extravasation and the Foley’s catheter was removed. Repeated serum creatinine results showed the resolution of the kidney injury, and the serum creatinine levels returned to normal.

## Discussion

Mechanism of development of urinothorax

Pleural effusion typically occurs when the rate of fluid accumulation exceeds the rate of fluid removal by the pleural lymphatics [4]. Urine can accumulate in the pleural space either directly or indirectly [4]. It occurs directly by moving into the pleural space down the pressure gradient between the abdomen and the pleural space through diaphragmatic pores. Another direct mechanism is through the development of a urinoma - urine collection outside the urinary tract in the retroperitoneum [5] - which can directly rupture into the pleural space, causing urinothorax. Urinothorax can develop indirectly when urine moves via lymphatic communication between the retroperitoneum and the pleural space. In the absence of a traumatic leak in the GU tract, unilateral kidney obstruction is thought not to cause urinothorax as long as the other kidney is functioning normally. In cases of unilateral obstruction with normal kidney function and the presence of urinothorax, the proposed mechanism is obstruction, which causes traumatic injury to the GU tract and leads to the development of urinothorax [2].

Pleural fluid analysis (PFA)

Urinothorax is one of the causes of transudative pleural effusion. This is not always the case, however, as it sometimes coexists with other processes, such as infection or malignancy, which causes the overall pleural effusion to be exudative. If the pleural effusion results only from urinothorax, the aspirated fluid will be clear and yellow with a urine smell [2] and will be considered transudative according to Light’s criteria. As reported by Austin et al. in 2017 [3], among the 57 cases of urinothorax that were published in the English literature between 1960 and 2016, the pleural fluid was transudative in 28 (49%), exudative in 13 (23%), and not classified in 16 (28%). In cases with hematuria and urinothorax, the pleural fluid can be exudative due to the passage of plasma protein into the fluid [2]. Pleural fluid pH is usually <7.4 and the glucose level is the same as in the serum unless there is a concomitant infection [4]. The most pathognomonic feature of the PFA is the presence of a high creatinine level and a PF/S Cr ratio >1, which is confirmatory [1,6]. The creatinine concentration of pleural fluid has been reported to be inversely related to the time of thoracentesis: the longer the period between the onset of symptoms and thoracentesis, the lower the pleural creatinine level. This is most likely secondary to a dilution effect from increasing pleural effusion [3]. PFA reports of LDH levels in urinothorax vary from low to high. The most likely explanation for an elevated LDH level is an underlying urinary process causing cellular disruption, as LDH is known to be a nonspecific marker of cellular disruption anywhere along the urinary tract. [3]. Glucose levels are usually low [5] because they are typically low in urine [2]. PFA commonly shows few nucleated cells. The cell count and the differential count will, however, vary if there is a concomitant infection [2].

Diagnosis

The clinical presentation of urinothorax usually starts with dyspnea, which occurs after pleural fluid accumulation becomes significant [4]. Urinothorax should be suspected in patients with pleural effusion and a concomitant GU condition, including obstructive uropathy, or a recent GU procedure. PFA will show paucicellular, transudative fluid with a PF/S Cr ratio >1 and pH <7.4. If PFA suggests urinothorax, appropriate abdominal imaging, including ultrasound (US) and CT, is recommended to further identify the site of pathology and to confirm the presence of urinothorax. Of note, exudative PFA should not exclude a diagnosis of urinothorax, as multiple processes can coexist. In rare cases in which urinothorax is suspected but cannot be confirmed by PFA and conventional imaging, such as US and CT, radionuclide scintigraphy and single-photon emission CT with technetium-99m diethylenetriaminepentaacetic acid (99mTC-DTPA) can be used [3]. 99mTC-DTPA is normally excreted by the kidneys. If the subsequent renography scan shows the presence of this radioisotope in the pleural space, it signifies that urine is reaching it and a diagnosis of urinothorax can be confirmed.

Management

To successfully treat urinothorax, a multidisciplinary team, including a urologist and pulmonologist, is needed. The mainstay of urinothorax treatment is to relieve the obstruction with a nephrostomy tube or a Foley’s catheter or to repair the GU tract. Treating the underlying GU pathology will resolve the urinothorax and prevent its recurrence [2,4,6-7]. Thoracentesis is usually performed for therapeutic and diagnostic purposes. There is no need for thoracic surgical intervention because, as reported by Austin et al. [4], of the 57 cases of urinothorax, none required thoracic surgical intervention. Pleurodesis is also ineffective [2].

## Conclusions

Urinothorax is a rare diagnosis that can be missed. It should be included in the differential diagnosis when a patient presents with pleural effusion with a PF/S Cr ratio >1 and a recent GU disease or procedure. A multidisciplinary team, including a pulmonologist and a urologist, is usually required for treatment. After the GU disease is treated, the urinothorax should resolve.
